# Nobiletin Inhibits Angiogenesis by Regulating Src/FAK/STAT3-Mediated Signaling through PXN in ER^+^ Breast Cancer Cells

**DOI:** 10.3390/ijms18050935

**Published:** 2017-04-30

**Authors:** Nipin Sp, Dong Young Kang, Youn Hee Joung, Jong Hwan Park, Wan Seop Kim, Hak Kyo Lee, Ki-Duk Song, Yeong-Min Park, Young Mok Yang

**Affiliations:** 1 Department of Pathology, School of Medicine, Institute of Biomedical Science and Technology, Konkuk University, Seoul 05029, Korea; nipinsp@gmail.com (N.S.); kdy6459@naver.com (D.Y.K.); adada76@nate.com (Y.H.J.); nihpark@yahoo.com (J.H.P.); wskim@kuh.ac.kr (W.S.K); 2 Department of Animal Biotechnology, Chonbuk National University, Jeonju 54896, Korea; breedlee@empal.com (H.K.L.); kiduk.song@gmail.com (K.-D.S.); 3 Department of Immunology, School of Medicine, Konkuk University, Chungju 27478, Korea; immun3023@kku.ac.kr

**Keywords:** nobiletin, angiogenesis, FAK, Src, STAT3, PXN

## Abstract

Tumor angiogenesis is one of the major hallmarks of tumor progression. Nobiletin is a natural flavonoid isolated from citrus peel that has anti-angiogenic activity. Steroid receptor coactivator (Src) is an intracellular tyrosine kinase so that focal adhesion kinase (FAK) binds to Src to play a role in tumor angiogenesis. Signal transducer and activator of transcription 3 (STAT3) is a marker for tumor angiogenesis which interacts with Src. Paxillin (PXN) acts as a downstream target for both FAK and STAT3. The main goal of this study was to assess inhibition of tumor angiogenesis by nobiletin in estrogen receptor positive (ER^+^) breast cancer cells via Src, FAK, and STAT3-mediated signaling through PXN. Treatment with nobiletin in MCF-7 and T47D breast cancer cells inhibited angiogenesis markers, based on western blotting and RT-PCR. Validation of in vitro angiogenesis in the human umbilical vein endothelial cells (HUVEC) endothelial cell line proved the anti-angiogenic activity of nobiletin. Electrophoretic mobility shift assay and the ChIP assay showed that nobiletin inhibits STAT3/DNA binding activity and STAT3 binding to a novel binding site of the *PXN* gene promoter. We also investigated the migration and invasive ability of nobiletin in ER^+^ cells. Nobiletin inhibited tumor angiogenesis by regulating Src, FAK, and STAT3 signaling through PXN in ER^+^ breast cancer cells.

## 1. Introduction

Breast cancer is associated with the second highest mortality rate worldwide [1]. Among the many causes of breast cancer, environmental factors play an important role [2]. Different factors regulate tumor progression, such as uncontrolled cell proliferation, induction of the cell cycle, and loss of apoptosis-inducing ability. Estrogen receptor (ER) has a significant role in breast cancer and is considered a predictive marker for endocrine therapy in breast cancer patients [3].

Tumor angiogenesis is considered a key hallmark of tumor progression. Formation of new blood vessels occurs as compensation for a lack of blood supply to provide sufficient oxygen for tumor development [4]. Tumors also trigger blood vessel formation by secreting several growth factors that can induce capillary growth. Vascular endothelial growth factor (VEGF) is the major growth factor in tumor angiogenesis, and basic fibroblast growth factor (bFGF) and epidermal growth factor (EGF) interact with VEGF during this process [5].

Steroid receptor coactivator (Src) and focal adhesion kinase (FAK) are intracellular tyrosine kinases that play an important role in tumor angiogenesis. FAK is associated with the EGF receptor (EGFR), and EGF-associated cell migration depends on FAK [6]. FAK aids in intracellular signaling events, and its absence is linked to alterations in morphology as well as in cell migration ability [7]. Src is a proto-oncogene that plays a vital role in cell motility, cell proliferation, and survival, which underlies its role in tumor angiogenesis [8]. It acts as a starting point for various biochemical pathways and transmits signals to downstream molecules [9].

FAK can regulate tumor angiogenesis as well as cell survival by forming a complex with Src through phosphorylation of paxillin (PXN) [10]. PXN is a focal adhesion–related protein that interacts with the Src–FAK complex during tumor angiogenesis. Signal transducer and activator of transcription 3 (STAT3) is an oncogene that takes part in tumor progression and metastasis. STAT3 plays a vital role in angiogenesis by transcriptional activation of VEGF expression, which is induced by Src and EGFR [11,12]. PXN is a downstream target for FAK, and the FAK–PXN interaction regulates cell migration and metastasis [13]. STAT3 acts as a transcription factor for PXN, and an absence of PXN leads to the mislocalization of activated STAT3. Src is required for this activation, which helps in localization and phosphorylation of STAT3 [14].

Targeting specific pathways included in vessel formation during tumor angiogenesis depends on the mode of action of the cancer drug. Cancer treatment with a natural substance can be a convenient approach because of high efficacy and potentially limited side effects. Nobiletin (5,6,7,8,3′,4′-hexamethoxyflavone) is a flavonoid isolated from citrus peels. It is considered an anti-inflammatory and immunomodulatory drug [15], increases α-amino-3-hydroxy-5-methyl-4-isoxazolepropionic acid (AMPA) receptor activity and long-term potentiation in cell culture [16], and can inhibit matrix metalloproteinases (MMPs) [17]. Various studies have shown that nobiletin has anti-cancer activity [18,19,20,21].

In this work, we evaluated the ability of nobiletin to inhibit tumor angiogenesis by regulating Src/FAK/STAT3 signaling through PXN in ER^+^ breast cancer cells. In addition, we identified a novel binding site for STAT3 in the *PXN* gene promoter.

## 2. Results

### 2.1. Nobiletin Inhibits Cell Proliferation of Various Breast Cancer Cell Lines and an Endothelial Cell Line

**Figure 1:**
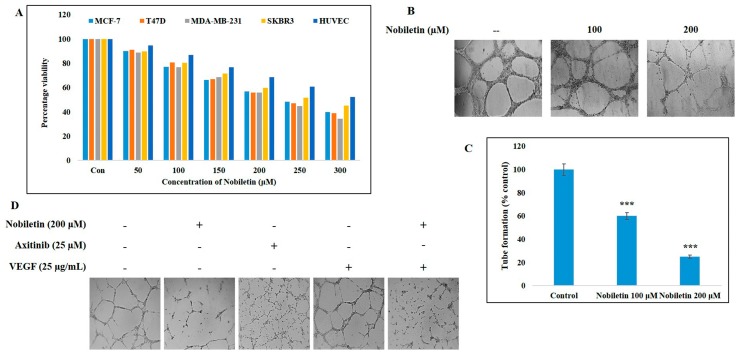
Nobiletin inhibited cell proliferation of various cell lines and vascular endothelial growth factor (VEGF)-dependent in vitro angiogenesis. (**A**) MTT assay showing the cell proliferation results for MCF-7, T47D, MDA-MB-231, SKBR3, and HUVEC cell lines under nobiletin treatment for 24 h; (**B**) Inhibition of angiogenesis by nobiletin using the in vitro angiogenesis assay in HUVEC cells; (**C**) Graphical representation of the in vitro angiogenesis assay showing the relative inhibition of tube formation. Statistical analyses were conducted using Student’s *t*-tests (*** *p* < 0.001); (**D**) In vitro angiogenesis assay in HUVEC cells showing the inhibition of angiogenesis after treatment with 200 μM nobiletin, 25 μM axitinib, and pre-treatment with 25 μg/mL recombinant human VEGF followed by nobiletin treatment.

The cell proliferation inhibition ability of nobiletin was evaluated by 3-(4,5-dimethylthiazol-2-yl)-2,5-diphenyltetrazolium bromide (MTT) assay. The ER^+^ cell lines MCF-7 and T47D, the triple-negative breast cancer cell lines MDA-MB-231, Her2^+^, and SKBR3, and the endothelial cell line human umbilical vein endothelial cells (HUVEC) were treated with various concentrations of nobiletin, and cell proliferation was assessed by comparison with non-treated control cells (Figure 1A). The results suggested that nobiletin inhibited cell proliferation more in MDA-MB-231 cells and less in the HUVEC line. From the data, we identified an IC_50_ dosage of 200 μM nobiletin in both MCF-7 and T47D cell lines, which was used for further studies.

### 2.2. Nobiletin Inhibits VEGF-Dependent In Vitro Angiogenesis

To determine the angiogenesis inhibition ability of nobiletin, we used an in vitro angiogenesis assay with nobiletin at 100 and 200 μM (Figure 1B). Nobiletin at 200 μM significantly inhibited tube formation in the extracellular matrix (Figure 1C). To check VEGF-dependent angiogenesis, we used recombinant human VEGF (25 μg/mL) and the VEGF inhibitor axitinib (25 μM) along with 200 μM nobiletin for assaying in vitro angiogenesis. A concentration of 25 μM of axitinib was considered as the IC_50_ dosage which inhibits VEGF activity so that this concentration can be used to compare VEGF inhibition ability of nobiletin. The results suggested that nobiletin inhibited VEGF-dependent angiogenesis (Figure 1D).

### 2.3. Nobiletin Inhibits EGFR Activity and Downregulates Src/FAK/STAT3 Signaling

**Figure 2:**
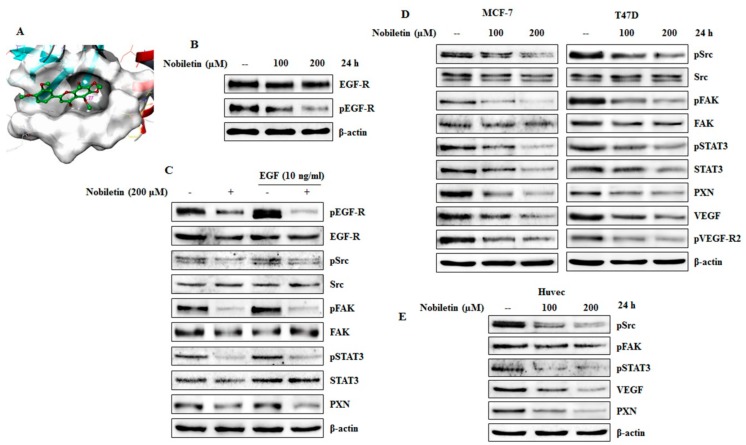
Nobiletin inhibited EGFR activity and Src/FAK/STAT3 signaling. (**A**) Binding of nobiletin (PubChem ID: 72344) to the ATP-binding domain of EGF-R (PDB ID: 2GS2) determined through molecular docking using Autodock vina; (**B**) Western blotting analysis of MCF-7 cells showing that nobiletin inhibited phosphorylated EGFR expression in a concentration-dependent manner; (**C**) MCF-7 cells were pre-treated with recombinant EGF (10 ng/mL) for 10 min and then treated with 200 μM nobiletin followed by isolation of protein and analysis using western blotting; (**D**) Western blotting analysis showing the inhibition of Src/FAK/STAT3 signaling in ER^+^ breast cancer cells after treatment with nobiletin for 24 h; (**E**) Src/FAK/STAT3 signaling inhibition by nobiletin in HUVEC cells by western blotting analysis.

To identify the molecular mechanisms underlying the anti-angiogenic ability of nobiletin, we tested the binding ability of nobiletin with EGFR. Molecular docking was performed with an autodock vina platform. Nobiletin docked with the ATP binding site of EGF-R and the result showed direct binding of nobiletin with the EGFR (Figure 2A). Then we conducted western blotting analysis of EGFR and phospho EGFR expression with nobiletin treatment. The expression of phosphorylated EGFR was downregulated by nobiletin (Figure 2B). Inhibition of EGFR receptor activity by nobiletin was confirmed by using recombinant human EGF (10 ng/mL). Western blotting analysis showed an inhibition of EGFR binding and of the downstream molecules Src, FAK, STAT3, and PXN (Figure 2C). To examine the effect of nobiletin on the angiogenic molecules in the Src/FAK/STAT3 signaling cascade, a dose-dependent analysis was carried out in ER^+^ breast cancer cell lines. The results indicated a downregulated expression pattern of these molecules along with the expression of PXN (Figure 2D). These findings suggested that nobiletin inhibits angiogenesis through these signaling mechanisms. Western blotting analysis of Src/FAK/STAT3 signaling in HUVEC cells also confirmed the anti-angiogenic activity of nobiletin (Figure 2E).

### 2.4. VEGF and Concentration-Dependent Inhibition of Src/FAK/STAT3 Signaling and Downregulation of Angiogenic Factors by Nobiletin

**Figure 3:**
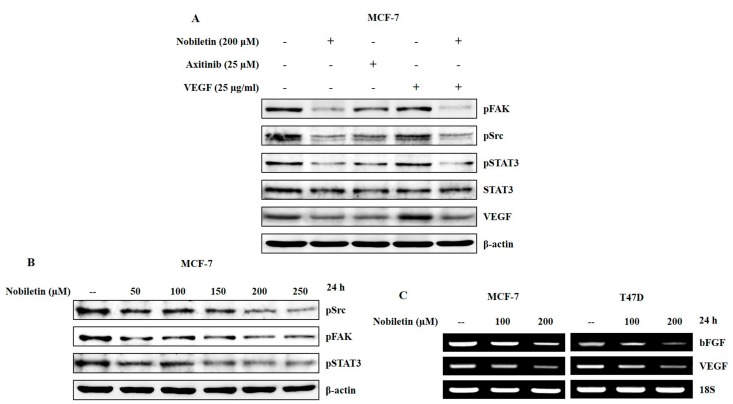
Nobiletin inhibited VEGF-dependent Src/FAK/STAT3 signaling and angiogenic factors. (**A**) Western blotting analysis in MCF-7 cells showing a downregulation of Src/FAK/STAT3 signaling after treatment with 200 μM nobiletin, 25 μM axitinib, and pre-treatment with 25 μg/mL recombinant human VEGF followed by treatment with nobiletin; (**B**) Concentration-dependent inhibition of Src/FAK/STAT3 signaling by nobiletin using western blotting analysis; (**C**) RT-PCR analysis showing the inhibition of angiogenic factors after treatment with nobiletin for 24 h.

To confirm the role of Src/FAK/STAT3 in the anti-angiogenic activity of nobiletin, we used recombinant human VEGF and axitinib to treat MCF-7 cells and compared the results with those of nobiletin-treated cells. Nobiletin with VEGF and axitinib decreased the expression levels of Src, FAK, and STAT3 in VEGF-treated cells compared to cells treated with nobiletin alone (Figure 3A). A concentration-dependent analysis of these signaling molecules showed a downregulation in their expression pattern with increasing concentrations of nobiletin (Figure 3B). To elucidate the expression levels of the angiogenic factors VEGF and bFGF, we conducted RT-PCR analysis of RNA isolated from both MCF-7 and T47D with or without nobiletin treatment. The results also confirmed the inhibition of angiogenic factors at the transcriptional level (Figure 3C).

### 2.5. Nobiletin Inhibits Nuclear Translocation and DNA Binding Activity of STAT3

**Figure 4:**
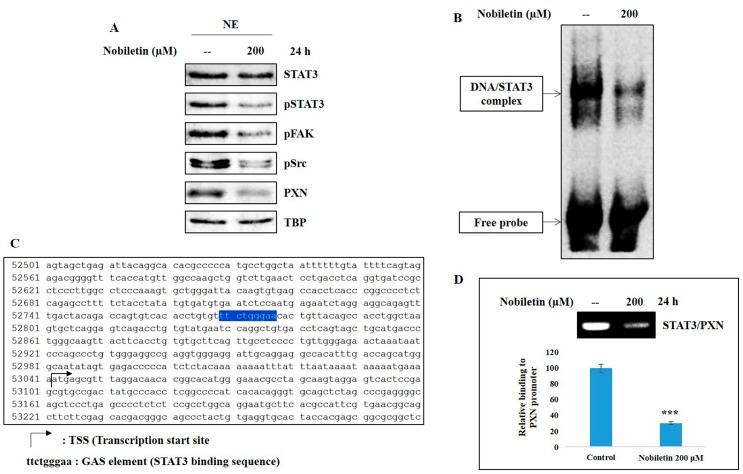
Nobiletin inhibited DNA binding activity and gene promoter binding of STAT3. (**A**) Western blotting analysis of MCF-7 nuclear proteins showing the inhibition of Src/FAK/STAT3 signaling and PXN; (**B**) EMSA analysis showing inhibition of the DNA binding activity STAT3 to the GAS element; (**C**) Sequence of the human *PXN* gene promoter (available online: https://www.ncbi.nlm.nih.gov/nuccore/NG_029820.1?from=5001&to=60333&report=genbank). A GAS element (ttctgggaa) present in the *PXN* gene (nucleotide sequence 52769-52777) is highlighted; (**D**) ChIP assay analysis showing the complex formation of STAT3/PXN and inhibition by nobiletin. The relative DNA binding of STAT3 to the PXN gene promoter was expressed as a percentage of the control. Statistical analysis was performed using Student’s *t*-tests (*** *p* < 0.001).

To affect transcription, phosphorylated STAT3 must be translocated to the nucleus and bind to the PXN gene promoter. Nuclear translocation was analyzed by isolating nuclear extracts from MCF-7 cells pretreated with nobiletin. Western blotting analysis of nuclear extracts showed a decrease in the expression levels of phosphorylated Src, FAK, and STAT3 along with PXN in nobiletin-treated cells (Figure 4A). The DNA binding activity of STAT3 to the gamma interferon activation site (GAS) element was analyzed by electrophoretic mobility shift assay (EMSA), which showed a downregulation of the DNA/STAT3 complex in nobiletin-treated cells compared to untreated control cells (Figure 4B). This binding suggested the need to determine the binding site of STAT3 in the *PXN* gene promoter region. We discovered the GAS element (ttctgggaa) in the *PXN* gene promoter, a binding site for STAT3 to initiate transcription (Figure 4C). We then confirmed this binding site using ChIP assay. The results showed an expression of the STAT3/PXN complex in the GAS element of the *PXN* gene promoter, which was confirmed by RT-PCR analysis (Figure 4D). Nobiletin inhibited the expression level of the STAT3/PXN complex, which indicated that nobiletin acts through STAT3 signaling.

### 2.6. Nobiletin Inhibits PXN Expression in a STAT3-Dependent Manner

**Figure 5:**
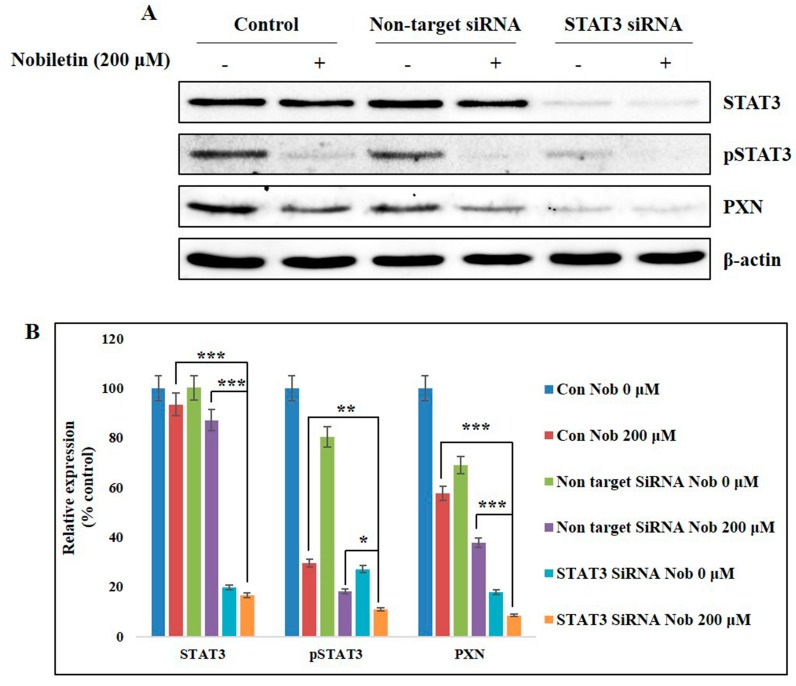
Nobiletin inhibited STAT3-dependent PXN expression. (**A**) On-target inhibition of STAT3 in MCF-7 cells by siRNA and expression analysis of STAT3 and PXN after treatment with nobiletin were performed using western blotting; (**B**) Relative expression of STAT3, pSTAT3, and PXN proteins. Statistical analysis was conducted using Student’s *t*-tests (* *p* < 0.05, ** *p* < 0.01, *** *p* < 0.001).

To identify the role of STAT3 in PXN expression, we used an siRNA gene-silencing method. First, STAT3 was knocked down with specific STAT3 siRNA, followed by treatment with nobiletin for 24 h. The results showed a complete inhibition of STAT3 expression in STAT3 siRNA cells (Figure 5A). Phosphorylated STAT3 and PXN expression levels also decreased in siSTAT3. Nobiletin again decreased the expression levels of phospho STAT3 and PXN. The relative expression of PXN and phosphorylated STAT3 indicated the involvement of STAT3 in PXN expression (Figure 5B).

### 2.7. Nobiletin Inhibits Migration and Cellular Invasive Potential

**Figure 6:**
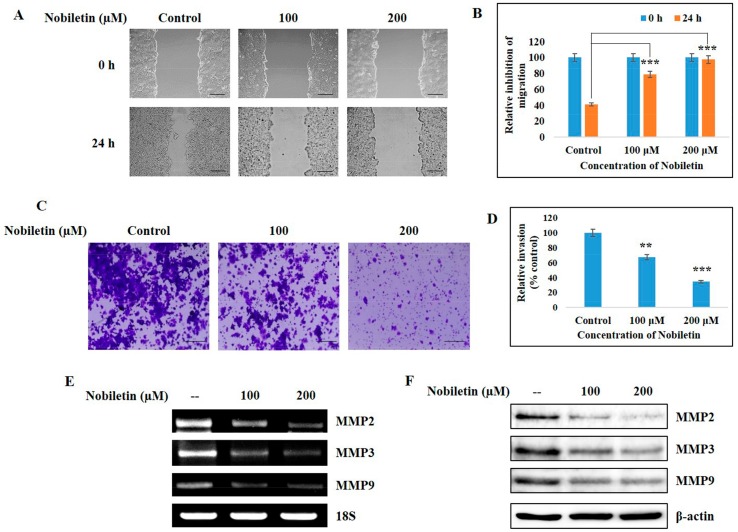
Nobiletin inhibited migration, cell invasion abilities, and MMP expression. (**A**) Wound-healing assay assessing the migration inhibition ability of nobiletin in MCF-7 cells for 24 h. Scale bars: 50 μm; (**B**) Relative inhibition of migration by nobiletin. Statistical analysis was conducted using ANOVA (*** *p* < 0.001); (**C**) Matrigel invasion assay showing the invasion inhibition by nobiletin in MCF-7 cells for 24 h. Scale bars: 100 μm; (**D**) Relative invasion of MCF-7 cells after treatment with nobiletin. Statistical analysis was performed using ANOVA (** *p* < 0.01, *** *p* < 0.001); (**E**) RT-PCR analysis of MMP2, MMP3, and MMP9 after treatment with nobiletin in MCF-7 cells for 24 h; (**F**) Western blotting analysis showing the inhibition MMP2, MMP3, and MMP9 by nobiletin in MCF-7 cells for 24 h.

Migration and invasion are considered as key regulation processes in the progression of tumor angiogenesis. Thus, we analyzed the inhibition ability of nobiletin against migration and invasion processes. Figure 6A shows the results of a wound-healing assay using MCF-7 cells for a treatment period of 24 h. Nobiletin inhibited the migration ability of MCF-7 cells in a dose-dependent manner (Figure 6B). The invasive inhibition ability of nobiletin was determined using a Matrigel invasion assay (Figure 6C), and these results also showed a decrease in the relative expression of invaded cells upon nobiletin treatment (Figure 6D). This invasion process mainly depended on the release of MMPs. Nobiletin downregulated the expression of MMP2, MMP3, and MMP9 at both the transcriptional (Figure 6E) and translational levels (Figure 6F). These results also indicated a metastasis inhibition ability of nobiletin in ER^+^ breast cancer cells.

## 3. Discussion

Treatment of cancer with natural compounds holds potential for a possible lack of side effects and good efficacy [22,23]. Nobiletin is known to exert anti-cancer activity in various cancers, acting against cell proliferation and possibly leading to cell cycle arrest or apoptosis. It has already been proved that nobiletin has anti-angiogenic ability against various cancers [24,25]. Here we checked the pathway responsible for the anti-angiogenic activity of nobiletin in ER^+^ breast cancer cells. Firstly, we checked the cell proliferation inhibition ability of nobiletin in different breast cancer cell lines as well as in a normal endothelial cell line (Figure 1A). A highly metastatic, triple-negative breast cancer cell line, MDA-MB-231, showed more inhibition in cell proliferation upon nobiletin treatment when compared with other cell lines. Nobiletin also inhibited the cell proliferation of the Her2^+^ breast cancer cell line SKBR3, the ER^+^ breast cancer cell lines MCF-7 and T47D, and endothelial cell line HUVEC. This outcome clearly indicated that nobiletin inhibits cell proliferation by targeting signaling molecules other than ER, progesterone receptor, or Her2/neu.

Control over tumor angiogenesis is crucial for anti-cancer therapy because this angiogenesis leads to tumor progression and metastasis [26]. VEGF has been considered as a key endothelial cell—specific signaling factor needed for tumor angiogenesis [27]. Nobiletin inhibited VEGF-dependent in vitro angiogenesis in ER^+^ breast cancer and endothelial cells (Figure 1B,D). Here we dissected the signaling cascade that is responsible for the anti-angiogenesis ability of nobiletin. Src and FAK are the two signaling molecules that have a significant role in tumor angiogenesis. Src induces angiogenesis by promoting VEGF expression [28], and FAK plays an important role in endothelial cell proliferation, migration, and survival [29]. PXN acts as a downstream target of Src/FAK signaling [30] and also plays the same role with STAT3. Here, Src regulated the expression of both FAK and STAT3, which then regulated the expression of PXN. Inhibition of these signaling molecules by nobiletin in ER^+^ breast cancer cells and HUVEC cells suggested that its anti-angiogenic ability may arise through these signaling molecules (Figure 2D,E). With a VEGF inducer and inhibitor, however, we showed that nobiletin inhibited tumor angiogenesis by regulating Src/FAK/STAT3 through PXN (Figure 3A). We confirmed these results with an EGF inducer by inhibiting the specific molecules by nobiletin (Figure 2B,C). Inhibition of the angiogenic factors bFGF and VEGF at the translational level by nobiletin also confirmed its anti-angiogenic ability (Figure 3C). As nobiletin inhibited general tumor angiogenesis in vitro by mediating Src/FAK/STAT3 signaling, in vivo experimentation is very important to confirm this signaling pathway.

Studies have already shown that STAT3 activity upregulates the expression of VEGF and thus tumor angiogenesis [11]. To understand the role of STAT3 through PXN in angiogenesis, we examined the nuclear translocation and DNA binding activity of STAT3. The results suggested that nobiletin inhibited the nuclear translocation of phosphorylated STAT3, Src, and FAK and the DNA binding activity of STAT3, as analyzed by EMSA (Figure 4A,B). STAT3 can bind the GAS element of its target gene promoter [31]. Here we assumed that STAT3 binds to the GAS element in the *PXN* gene promoter region. We characterized the GAS element in the *PXN* gene promoter region (ttctgggaa) by using the *PXN* gene database from NCBI GenBank (Figure 4C) and confirmed the EMSA result of STAT3 binding to the GAS element of the *PXN* gene promoter. For further evidence, we conducted a ChIP assay with the specific primers targeting the *PXN* gene promoter region that includes the GAS element and confirmed the formation of the STAT3/PXN complex. In addition, nobiletin inhibited the expression of these complexes, confirming its ability to inhibit DNA binding activity (Figure 4D). The role of STAT3 with PXN was also confirmed after silencing the expression of STAT3 (Figure 5). These results clearly demonstrated the inhibition of angiogenesis by regulating STAT3 through PXN. Altogether, nobiletin inhibited the expression of PXN through Src/FAK or Src/STAT3, which accounts for its anti-angiogenic ability.

MMPs are major contributors in tumor angiogenesis, and their inhibition blocks new capillary formation [32]. These MMPs thus play a vital role in tumor cell migration and invasion [33]. Here we found that nobiletin can inhibit the tumor migration and invasion ability of ER^+^ breast cancer cells (Figure 6A,C), which also helps to explain the anti-angiogenic activity of nobiletin. Inhibition of MMPs by nobiletin at both the transcriptional and translational levels demonstrated the angiogenic inhibition capacity of nobiletin. Inhibition of migration and invasion also suggests the inhibition of metastasis by nobiletin.

Altogether, nobiletin inhibited general angiogenesis through mediating Src/FAK/STAT3 signaling molecules in vitro. However, further experimentation using breast cancer xenograft model is much needed to confirm the role of these signaling molecules during the angiogenesis inhibition by nobiletin. The importance of in vivo experiments in this work is to confirm and establish a molecular pathway responsible for the treatment of tumor angiogenesis by nobiletin. In vivo study also provides an idea about tumor metastasis. Nobiletin already inhibited cell migration and invasion in vitro, which clearly pointing towards metastasis inhibition by nobiletin through mediating Src/FAK/STAT3 and PXN signaling.

In conclusion, nobiletin inhibited tumor angiogenesis by regulating Src/FAK/STAT3-mediated signaling through PXN in ER^+^ breast cancer cells. We discovered a novel binding site for STAT3 in the *PXN* gene promoter, which is responsible for tumor angiogenesis inhibition by nobiletin. However, further analysis using in vivo experimentation may provide much conclusive evidence which confirms the role of Src/FAK/STAT3-mediated signaling in angiogenesis inhibition by nobiletin.

## 4. Materials and Methods

### 4.1. Antibodies and Reagents

Human breast adenocarcinoma, MCF-7, and T47D cell lines were purchased from South Korean Cell Bank (Seoul, Korea). RPMI-1640, β-actin antibody and Imprint chromatin immunoprecipitation assay kit was purchased from Sigma Chemical (St. Louis, MO, USA). Penicillin-streptomycin solution and fetal bovine serum (FBS) were purchased from Hyclone (South of Logan, UT, USA). 0.05% trypsin-ethylenediaminetetraacetic acid was purchased from Gibco-BRL (Grand Island, NY, USA). In vitro angiogenesis kit and Jak2 antibody were obtained from Millipore (Billerica, MA, USA). EGFR, p-EGFR, Src, FAK, STAT3, p-STAT3, VEGF, VEGF-R2, PXN, MMP2, MMP3, MMP9 antibodies, and secondary antibodies (goat anti-mouse and rabbit immunoglobulin G IgG-horseradish peroxidase) were obtained from Santa Cruz Biotechnology (Santa Cruz, CA, USA). p-Src, p-FAK, p-VEGF-R2, p-Jak2 antibody were purchased from Cell Signaling Technology (Beverly, MA, USA). The WesternBright ECL HRP substrate detection solution was purchased from Advansta Inc. (Menlo Park, CA, USA). Restore western blot stripping buffer and NE-PER kits were purchased from Pierce (Rockford, IL, USA). RNeasy mini kits and Qiaprep spin miniprep kits were purchased from Qiagen (Hilden, Germany). Reverse transcriptase-polymerase chain reaction (RT-PCR) premix kits and VEGF, bFGF, MMP2, MMP3, MMP9, 18s primers for RT-PCR were synthesized by Bioneer (Daejon, Korea). Electrophoretic mobility shift assay (EMSA) kit and oligonucleotide probes (STAT3) were obtained from Promega Corp (Madison, WI, USA).

### 4.2. Cell Culture and Treatment

ER^+^ cell lines, MCF-7 and T47D cell lines, triple negative cell line, MDA-MB-231, Her2^+^ cell line, and SKBR3 were cultured and maintained in RPMI-1640 medium containing 10% FBS, 100 U/mL penicillin at 37 °C in 5% CO_2_. The endothelial cell line HUVEC was maintained in EBM-2 endothelial growth basal media. The cells were placed in airtight chambers (Nu Aire, Plymouth, MN, USA). At the beginning of each experiment, the cells were counted depending on the experiment and were re-suspended in the medium. Cells were treated with nobiletin at different concentrations according to the experiments.

### 4.3. Cell Proliferation Inhibition

Cell viability was assayed by measuring blue formazan that was metabolized from MTT by mitochondrial dehydrogenase. The cells were re-suspended in the medium one day before drug treatment, at a density of 3 × 10^3^ cells per well in 96-well culture plates. Liquid medium was replaced with fresh medium containing dimethyl sulfoxide (DMSO) for control (vehicle). Cells (MCF-7, T47D, MDA-MB-231, SKBR3 and HUVEC) were incubated with various concentrations of nobiletin. MTT (5 mg/mL) was added to each well and incubated for 4 h at 37 °C. The formazan product formed was dissolved by adding 200 μL DMSO to each well, and the absorbance was measured at 570 nm on an Ultra Multifunctional Microplate Reader (TECAN, Durham, NC, USA). All measurements were performed in triplicate, and were repeated at least three times.

### 4.4. In Vitro Angiogenesis Assay

ECMatrix was thawed at 4 °C overnight, and each well of pre-chilled 96-well plates were coated with 50 μL diluted ECMatrix and incubated at 37 °C for 1 h. A total of 150 μL of HUVEC cells (1 × 10^4^) with or without nobiletin was added to the solidified matrix and incubated at 37 °C for 12 h. Endothelial cell formation was observed using a microscope. Focus was placed on distinct areas and the tubes formed were counted.

### 4.5. Western Blotting

The MCF-7 and T47D cell lines were treated with nobiletin for 24 h. Whole cells were lysed on ice with radio immunoprecipitation lysis buffer containing phosphatase and protease inhibitors. Cells were disrupted and centrifuged at 15,000 rpm for 10 min at 4 °C to remove cellular debris. Protein concentrations were measured using the Bradford method. Equal amounts of proteins were resolved on sodium dodecyl sulfate-polyacrylamide gel electrophoresis (SDS-PAGE) and transferred onto nitrocellulose membrane. The blots were blocked for 1 h with 5% BSA. Membranes were probed overnight at 4 °C with a primary antibody followed by HRP-conjugated secondary antibodies. Detection was performed using the ECL Plus detection kit and an LAS-4000 imaging device (Fujifilm, Japan).

### 4.6. Reverse Transcription-PCR

Total RNAs were extracted using RNeasy Mini Kits (Qiagen, Hilden, Germany) and quantified spectrometrically at 260 nm. RT-PCR analysis for VEGF, bFGF, MMP2, MMP3, MMP9 and 18s RNAs was then performed. cDNA was synthesized from total RNA by RT at 42 °C for 1 h and 80 °C for 15 min using first-strand cDNA synthesis kits (Bioneer, Korea). PCR was conducted using cDNA. The primers used for the amplification are listed in the Table 1. PCR products were analyzed by 1% agarose gel stained with ethidium bromide.

### 4.7. EMSA

The DNA binding activity of STAT3 was assessed using EMSA, in which labeled double-stranded DNA was used as a DNA probe to bind active STAT3 proteins in nuclear extracts. Nuclear protein extracts were prepared with a nuclear extract kit (Panomics, AY2002). The EMSA experiment was performed by incubating a biotin-labeled transcription factor-STAT3 probe with nobiletin treated and untreated nuclear extracts. Proteins were resolved on a non-denaturing 6% PAGE gel (Bio-Rad, Korea). The proteins in the gel were transferred to a nylon membrane and detected using streptavidin-HRP and a chemiluminescent substrate.

### 4.8. ChIP Assay

A ChIP assay was performed using an Imprint Chromatin Immunoprecipitation Kit according to the manufacturer’s protocol. MCF-7 cells were fixed with 1% formaldehyde and quenched with 1.25 M glycine. After washing with PBS, the cells were suspended in nuclei preparation buffer and sonicated in shearing buffer under optimized conditions. This sheared DNA was diluted with dilution buffer (1:1 ratio) and 5 µL of diluted samples were removed as an internal control. The diluted supernatant was incubated with antibody (STAT3) in pre-coated wells for 90 min. Normal mouse IgG and anti-RNA polymerase II were used for the respective negative and positive controls. The unbound DNA was washed off with immunoprecipitation (IP) wash buffer and the bound DNA was collected by cross link reversal using DNA release buffer containing proteinase K. The released DNAs and the DNA from the internal controls were purified with GenElute Binding Column G. The DNA was then quantified using conventional PCR.

### 4.9. Wound Healing Assay

MCF-7 cells were cultured in 6-well plates at a concentration of 1 × 10^5^ cells/well in RPMI-1640 media and incubated for 24 h. After becoming a confluent monolayer, the cell layers were scratched with a 10 μL pipette tip and washed with PBS to remove cell debris. Cells were treated with 100 and 200 μM of nobiletin. Control cells were not treated. Wound edges were photographed at different time intervals using a microscope. The relative area of wound closure was measured using ImageJ software (NIH Image, Bethesda, MD, USA).

### 4.10. Matrigel Invasion Assay

The transwell invasion assay was performed with the help of Matrigel pre-coated, ready to use invasion chambers (BD Biocoat, Waltham, MA, USA). Cells suspended at 5 × 10^4^ were added to the inserts. The media containing nobiletin or without nobiletin were added to the receiver plate and the inserts were placed onto it. After a 24 h incubation in a humidified chamber at 37 °C, the cells that invaded to the apical surface of the inserts were resolved with crystal violet. The cells on the upper surface were removed using a cotton swab and the invaded cells were observed using a microscope. Focus was placed on four distinct areas and the cells were counted.

### 4.11. Small Interference RNA (siRNA) Analysis

MCF-7 cells (1 × 10^5^) were cultured on 6-well plates and grown to 60% confluence. The cells were then transfected with ON-TARGET plus SMARTpool siRNA targeting STAT3 or ON-TARGETplus non-targeting siRNA using DharmaFECT transfection reagent (Dharmacon, Chicago, IL, USA) according to the manufacturer’s instructions. Following transfection with this mixture for 24 h, nobiletin treatment was applied for an additional 24 h. Different areas were captured and the cells were counted.

### 4.12. Statistical Analysis

All the experiments were repeated three times and the results were expressed as the mean ± SEM. Groups were compared with the Student’s *t*-test or ANOVA. Statistical analyses were performed with the statistical analysis system (SAS) program.

## 5. Conclusions

These studies indicated that nobiletin inhibited tumor angiogenesis by modulating Src/FAK/STAT3 through PXN in ER^+^ breast cancer cells. We also found that STAT3 has a role in tumor angiogenesis through PXN and we discovered a new binding site for STAT3 in the PXN gene promoter, which plays an important role in inhibition of tumor angiogenesis by nobiletin.

## Figures and Tables

**Table 1:** RT-PCR primers, annealing temperature, product sizes and sequences for bFGF, VEGF, MMP2, MMP3, MMP9 and 18S genes.

Sl No	Gene	Annealing Temperature (°C)	Product Size (bp)	Sequence (5′–3′)
1	*bFGF*	58	498	Forward: gagaagagcgaccctcaca
				Reverse: tagctttctgcccaggtcc
2	*VEGF*	58	405	Forward: aggagggcagaatcatcacg
				Reverse: caaggcccacagggattttc
3	*18S*	58	490	Forward: agccttcggctgactggctgg
				Reverse: ctgcccatcatcatgacctgg
4	*MMP2*	53	665	Forward: gagttggcagtgcaatacct
				Reverse: gccatccttctcaaagttgt
5	*MMP3*	60	432	Forward: cctgctttgtcctttgatgc
				Reverse: tgagtcaatccctggaaagt
6	*MMP9*	58	455	Forward: cctgccagtttccattcatc
				Reverse: gccattcacgtcgtccttat
7	*PXN* (ChIP assay)	60	181	Forward: gcccctctcagagccttttc
				Reverse: gcagctactgaggtcacagc
